# Evaluación económica comparativa sobre terapias de reemplazo renal en Argentina, Costa Rica y Uruguay

**DOI:** 10.26633/RPSP.2021.119

**Published:** 2021-10-18

**Authors:** Santiago Torales, José Berardo, Santiago Hasdeu, María Paula Esquivel, Alfonso Rosales, Carlos Azofeifa, Jordan Salazar, Manuel Cerdas, Oscar Gianneo, Martín Esteche, Eliana Leguizamo, Alexandre Lemgruber, Mauricio Beltrán, Francisco Caccavo

**Affiliations:** 1 Dirección de Investigación en Salud Ministerio de Salud de la Nación Argentina Dirección de Investigación en Salud, Ministerio de Salud de la Nación, Argentina; 2 Universidad Nacional del Litoral Argentina Universidad Nacional del Litoral, Argentina.; 3 Universidad Nacional del Comahue Argentina Universidad Nacional del Comahue, Argentina.; 4 Caja Costarricense de Seguro Social Costa Rica Caja Costarricense de Seguro Social, Costa Rica.; 5 Hospital México San José Costa Rica Hospital México, San José, Costa Rica.; 6 Fondo Nacional de Recursos Uruguay Fondo Nacional de Recursos, Uruguay; 7 Organización Panamericana de la Salud Washington D.C. Estados Unidos de América Organización Panamericana de la Salud, Washington D.C., Estados Unidos de América.

**Keywords:** Enfermedad renal crónica, factores epidemiológicos, trasplante renal, diálisis, costo efectividad, Argentina, Costa Rica, Uruguay, Renal inssufficiency, chronic, epidemiologic factors, kidney transplantation, dialysis, cost effectiveness, Argentina, Costa Rica, Uruguay, Insuficiência renal crónica, fatores epidemiológicos, transplante de rim, diálise, custo-efetividade, Argentina, Costa Rica, Uruguai

## Abstract

**Objetivo.:**

Evaluar las diferencias de costos y prevalencia de las terapias de remplazo renal (TRR) como el trasplante, la diálisis peritoneal y la hemodiálisis en Argentina, Costa Rica y Uruguay, mediante estrategias costo-efectivas de difusión.

**Métodos.:**

Costos y prevalencia de principales financiadores y prestadores por país, y análisis de costo-efectividad mediante modelo de Markov a 5 años, evaluando estrategias de asignación de recursos expresadas por razón incremental de costo-efectividad en costo por año de vida ajustado por calidad.

**Resultados.:**

Se observa dispersión entre los países en el acceso y los valores prestacionales de TRR, que afectan su prevalencia y el punto de equilibrio monetario. Desde el punto de vista de los costos, es más eficiente promover la realización de trasplantes y de diálisis peritoneal, y desalentar la indicación de hemodiálisis, aunque la disponibilidad de cada TRR por país requirió evaluaciones particulares.

**Conclusiones.:**

Promover la realización de trasplantes ahorra costos, aunque los puntos de equilibrio variables requieren determinar diferentes umbrales de costo-efectividad por país. En Argentina y Uruguay, la administración de TRR mejoraría su eficiencia si se aumentan la cantidad de pacientes en diálisis peritoneal y las tasas de donación para trasplantes. En Costa Rica (donde hay tasas elevadas de trasplantes y margen presupuestario), la incorporación de técnicas dialíticas se ajusta por demanda e incidencia de pacientes con ERCT.

## Abstract (en)

**Objective.:**

Evaluate differences in the cost and prevalence of renal replacement therapies (RRTs) such as transplants, peritoneal dialysis, and hemodialysis in Argentina, Costa Rica, and Uruguay, based on cost-effective dissemination strategies.

**Methods.:**

Costs and prevalence obtained from the main financers and providers in each country; analysis of cost-effectiveness using a Markov model with a five-year horizon, evaluating resource allocation strategies for their incremental cost-effectiveness ratio expressed as quality-adjusted years of life.

**Results.:**

There is observed dispersion among countries in terms of access to and beneficial value of RRTs, affecting their prevalence and monetary breakeven point. From the cost standpoint, it is more efficient to promote transplants and peritoneal dialysis, and to discourage hemodialysis, although the availability of each RRT in each country required a specific evaluation.

**Conclusions.:**

Promoting transplants saves costs, but the variable breakeven points make it necessary to determine different cost-effectiveness thresholds for each country. In Argentina and Uruguay, RRTs would be more cost-effective with an increase in the number of patients in peritoneal dialysis and higher donation rates for transplants. In Costa Rica (where there is a high transplant rate and large budgetary margin), the use of dialysis is aligned with demand and with the incidence of patients with terminal chronic kidney disease.

## RESUMO

**Objetivo.:**

Avaliar as diferenças de custos e prevalência das terapias de substituição renal (TSR) como o transplante, a diálise peritoneal e a hemodiálise na Argentina, na Costa Rica e no Uruguai, mediante estratégias de difusão custo-efetivas.

**Métodos.:**

Foram avaliados custos e prevalência dos principais financiadores e prestadores por país, e realizada análise de custo-efetividade mediante modelo de Markov para 5 anos, avaliando estratégias de alocação de recursos expressas pela razão de custo-efetividade incremental por ano de vida ajustado por qualidade.

**Resultados.:**

Foi observada, entre os países, dispersão no acesso e nos valores prestacionais de TSR, afetando sua prevalência e o ponto de equilíbrio monetário. Do ponto de vista dos custos, é mais eficiente promover a realização de transplantes e de diálise peritoneal e desestimular a indicação de hemodiálise, embora a disponibilidade de cada TSR por país tenha exigido avaliações específicas.

**Conclusões.:**

Promover a realização de transplantes economiza custos, embora os pontos de equilíbrio variáveis requeiram a determinação de diferentes limiares de custo-efetividade por país. Na Argentina e no Uruguai, a administração de TSR melhoraria sua eficiência se a quantidade de pacientes em diálise peritoneal e as taxas de doação para transplantes aumentassem. Na Costa Rica (onde há taxas elevadas de transplantes e margem orçamentária), a incorporação de técnicas dialíticas é ajustada por demanda e incidência de pacientes com DRCT.

La enfermedad renal crónica terminal (ERCT) muestra una creciente prevalencia a nivel mundial y especialmente en las Américas (1), influenciada por mejoras en el acceso a la atención en los sistemas de salud de la Región y en la disponibilidad de terapias de reemplazo renal (TRR), cuyas principales modalidades son el trasplante (TX), la hemodiálisis (HD) y la diálisis peritoneal (DP) (2). Estos tratamientos requieren características específicas de capacitación profesional e infraestructura edilicia y tecnológica, se articulan en procesos de atención complejos y costosos, y constituyen un desafío permanente para el uso eficiente de los recursos sanitarios. Las TRR no permiten un uso alternativo y excluyente entre las modalidades, dado que un paciente puede requerir de manera secuencial algunas de ellas según su condición clínica y las posibilidades que le ofrece el sistema de salud. Dado que entre las diferentes modalidades existen diferencias marginales en ganancias de salud, su análisis de costo-efectividad (CE) requiere no solo una comparación sobre la eficiencia técnica, sino una evaluación del resultado sanitario obtenido desde una perspectiva de asignación de recursos en cuanto al acceso y difusión de las TRR (3). Por último, los resultados de CE dependen tanto de los precios relativos de las prestaciones como de su efectividad, a su vez condicionados con la forma de provisión y financiamiento de los servicios de salud.

En términos generales, los resultados clínicos ajustados por grupos de pacientes con ERCT no establecen diferencias en sobrevida y calidad de vida entre la HD y la DP, mientras que el TX ofrece beneficios en ambos indicadores (3), con una clara eficiencia luego del primer año (4-6). Sin embargo, las principales limitaciones para su expansión son: a) las tasas de donación limitada (la incidencia de pacientes en diálisis está en aumento en las Américas, con más de 180 000 personas en el 2016 en listas de espera, lo que supera ampliamente la disponibilidad de donantes) (7); b) muchos pacientes en diálisis nunca estarán en condiciones para recibir un TX; y c) la falta de capacidad en algunos sistemas de salud (oferta de especialistas y disponibilidad de tratamientos, incluidos los fármacos inmunosupresores y los equipos de apoyo) (8).

Así como muchos pacientes podrían no ser candidatos para recibir un TX, otros no serán candidatos a todas las modalidades de diálisis disponibles, especialmente las que requieren un alto grado de autonomía y participación (9,10). En este sentido, las distintas TRR (de estar disponibles) no pueden considerarse mutuamente excluyentes en términos de alocación de recursos, sino como alternativas de uso selectivo según la preferencia del paciente y las posibilidades de su entorno y el sistema de salud.

La bibliografía específica sobre estos aspectos es escasa en América Latina, y la transferibilidad de estudios de CE es aún motivo de discusión y debate. Bajo criterios de eficiencia, el enfoque actual se dirige hacia estrategias para promover la realización de TX y DP de acuerdo con las posibilidades del sistema de salud de cada país (8). Si bien el TX se encuentra en expansión (11) requiere del desarrollo de complejos procesos sanitarios accesorios (p. ej.: donación de órganos). Por otra parte, se evidencian grandes variaciones regionales en la prevalencia de DP, que oscilan entre 75% en México, 19% en Canadá y 8,2% en los Estados Unidos de América (12). Entre 1997 y 2008, el número de pacientes en DP aumentó en todo el mundo, con estabilización en los países en desarrollo, pero disminuyó 5,3% en países industrializados (2).

No todas las intervenciones (aún las potencialmente beneficiosas) pueden ser cubiertas presupuestariamente por los sistemas de salud sanitarios, incluso en las naciones más ricas (13, 14). Las evaluaciones económicas en salud (EES) buscan determinar el gasto requerido para obtener una unidad adicional de beneficio: la eficiencia de asignación determina cómo usar los recursos disponibles de manera efectiva para la atención de grupos de pacientes con distintos problemas de salud. De forma particular, para el análisis correcto de las TRR en pacientes ERCT desde la perspectiva de las EES, deben considerarse:

- Las posibilidades reales de implementar las alternativas de TRR.
- Su condición de alternativas “no mutuamente excluyentes”.
- La inclusión de las TRR como un proceso de oferta de servicios.
- La alta especificidad técnica como garantía de resultado de su aplicación.

La tecnología es una parte accesoria (de alto costo) de los dispositivos de atención, pero los resultados sanitarios (efecto clínico) dependen de variables más allá de la oferta tecnológica.

Estos condicionantes son más sensibles en países de medianos o bajos ingresos, y afectan los costos absolutos y relativos de las TRR (8). Existen factores relacionados con los tratamientos que contribuyen a estas variaciones, como la frecuencia de sesiones de HD por semana o los recambios por día en la DP, la fabricación local de insumos para DP, la infraestructura de las clínicas de HD, las barreras geográficas, el costo de inmunosupresores, la procuración de órganos y la existencia de centros especializados para realizar TX. El costo de desarrollar y mantener la infraestructura y la accesibilidad de los pacientes pueden ser factores clave, aunque difíciles de capturar y establecer en los estudios económicos (6-8). Sin embargo, y más allá de los costos absolutos, las diferencias en recursos económicos entre los países latinoamericanos, sus condiciones de pago prestacional o los incentivos financieros, influyen tanto en la llegada de los pacientes a las TRR como en la selección de estas, e incluso en la oferta de servicios y la formación de especialistas (11).

Con base en la bibliografía existente, es clara la superioridad del TX renal como TRR, con mejores resultados en la calidad de vida, la sobrevida de los pacientes y el mejor perfil de CE para los sistemas de salud (3). Con respecto a las técnicas dialíticas, la evidencia indica que la DP sería más eficiente, pero está infrautilizada. En los países en desarrollo, la DP suele tener el mismo costo, o menor, que otras modalidades de diálisis, y podría ser más atractiva si se optimiza mediante la fabricación local de insumos (9,10).

A fin de evidenciar este contexto genérico en el ámbito regional, y de impulsar la implementación de la estrategia y el Plan de acción de donación y acceso equitativo a trasplante de órganos, tejidos y células 2019-2030 de la Organización Panamericana de la Salud (OPS) (7), se propuso una EES completa que incluye el desarrollo de la estructura de costos directos y el relevamiento de probabilidades de acceso y transición en tres países de la Región. Se buscó indagar sobre el efecto de los precios relativos y la accesibilidad a la cobertura de TRR como condicionantes de la eficiencia, así como el efecto la organización del sistema de salud y los montos asimétricos de financiamiento en los resultados de CE de manera comparativa entre los países: dado que las TRR se desarrollan en el tiempo, se intentó definir el comportamiento de los costos a futuro, evaluando estrategias de difusión de TRR eficientes dentro de umbrales predefinidos en términos de razón incremental de costo efectividad (ICER) expresada como años de vida ajustados por calidad (AVAC).

## MATERIALES Y MÉTODOS

El trabajo se planteó como una evaluación económica completa, utilizando la perspectiva de los principales financiadores de las TRR en cada país. Para el acceso a la información relevante, la investigación se estructuró a partir de los ítems que se describen a continuación.

### Vías clínicas y costo de TRR por país

Se diseñaron vías clínicas para describir la utilización y los costos directos de las prestaciones relacionadas con las TRR para cada país. Las variables incluidas (sobre todo aquellas iterativas, como el número de sesiones en HD, los materiales y la frecuencia de recambios en DP, el uso de fármacos para el manejo de la anemia y el metabolismo óseo, y la evaluación, cirugía y seguimiento de TX) permitieron configurar módulos de cobertura. Para el presente estudio se tomaron los precios explícitos de los módulos existentes en los distintos sistemas de salud (en Argentina y Uruguay) o se construyeron de forma específica (para Costa Rica). De manera complementaria, se definieron clínicamente y se cotizaron la atención de complicaciones por TRR y las modalidades de inmunosupresión según la modalidad prestacional de cada país por protocolos y consultas de consenso con grupos focales de especialistas según el caso. Estos costos directos fueron tomados de los principales financiadores de TRR en cada país. En Costa Rica, se tomaron de la Caja Costarricense del Seguro Social (CCSS); en Uruguay, del Fondo Nacional de Recursos (FNR), y en Argentina se tomaron los valores ponderados del seguro social (Programa de Atención Médica Integral [PAMI] y las obras sociales nacionales y provinciales, que en conjunto cubren el 70% del financiamiento en TRR). Los valores se expresaron en moneda local y se convirtieron a dólares estadounidenses (U$S) al cambio oficial de cada país (junio del 2019). Los ítems principales considerados se describen a continuación:

**HD y DP:** módulo mensual (inclusiones y exclusiones, considerando fármacos) + complicaciones (acceso vascular, peritonitis e internaciones por episodios cardiovasculares o infecciosos).

**TX:** módulos pretrasplante + cirugía de trasplante + seguimiento postrasplante + complicaciones fuera de módulo (quirúrgicas, infecciosas, función retardada de comienzo o inducción adicional) + esquemas varios de inmunosupresión y tratamiento del rechazo agudo.

Para la inmunosupresión, el esquema basal inicial era tacrolimús + micofenolato mofetil (MFM) + prednisona, con dosis ajustadas a un paciente de 70 kg peso promedio, con reducciones semestrales en los 2 primeros años y dosis fija desde el tercer año. Se incluyeron ajustes por intolerancia al tacrolimús o al MFM (reemplazo por diana de rapamicina en células de mamífero [mTOR]). Las probabilidades se diagramaron en un árbol de decisión.

Para el rechazo agudo, a partir de la biopsia renal (con anatomopatología + microscopía electrónica + inmunofluorescencia), se ajustaron las tasas de variedad celular o humoral y sus tratamientos:

- Variedad celular Banff 0 y 1A: pulso de esteroides por vía intravenosa. Ante la falta de respuesta se recurre al tratamiento de segunda línea: nueva biopsia renal + antitimoglobulina + valganciclovir + 10 sesiones de plasmaféresis.
- Variedad celular Banff 1B a 3 y humoral: idéntico al tratamiento de segunda línea descrito arriba.

### Punto de equilibrio entre el trasplante y las técnicas dialíticas

A partir de los gastos anuales acumulados por cada TRR, se buscó determinar el momento en el que se neutralizan los mayores gastos ocasionados por el TX en el primer año con los potenciales ahorros de los años subsiguientes frente a las técnicas dialíticas, comparando el seguimiento a 3 años en los tres países.

### Prevalencia, probabilidades de transición y análisis de costo-efectividad

Se construyó un modelo de Markov considerando las TRR como estados de transición y se agregó la mortalidad como estado absorbente (15). Las prevalencias y las probabilidades de transición se obtuvieron de publicaciones (11) o ponderadas por registros oficiales retrospectivos de 2017 a 2019 de cada país. La estimación de escenarios se efectuó modificando de manera univariante los valores de prevalencia por método a partir de la referencia 2019 (caso base) mediante un análisis de sensibilidad determinístico simple aplicado solo a esta variable. Se calibró el modelo con una cohorte teórica de 1000 pacientes con ERCT hasta un horizonte temporal a 5 años y una tasa de descuento del 3%, que luego se aplicó al total de pacientes en TRR informado por cada país. Se obtuvieron resultados finales ajustados para: a) gasto total de TRR por país; b) mortalidad evitada; c) AVAC ganados; d) costo de cada muerte evitada y costo por AVAC de cada TRR; y e) costo por AVAC global de TRR. Cabe recordar que, en el caso de las TRR, su costo por AVAC ha sido considerado en muchos sistemas de salud como el umbral máximo de CE (16) y se tomó como referencia en este estudio para definir la ICER actual de cada país. Se generaron iteraciones del modelo modificando la prevalencia de cada TRR hasta encontrar las alternativas ubicadas bajo el umbral de CE definido y la dominancia sobre otras opciones. El modelo se diseñó en el programa Excel 2013^®^ de Microsoft.

### Definición de utilidades

Se calculó un valor promedio de AVAC por TRR a partir de diferentes estudios (17,18) que estimaron utilidades mediante el uso de diferentes escalas de calidad de vida: TX: 0,84; DP: 0,81 y HD: 0,72.

### Estimación de eficiencia en TRR por país

El gasto por país y per cápita en TRR se ponderó en relación con el costo anual por cada método y su distribución por prevalencia para determinar el umbral actual de CE. La ganancia relativa y el costo por AVAC se comparó entre las diferentes modalidades de TRR. Se utilizó como referencia adicional el producto bruto interno (PBI) per cápita de cada país correspondiente al mes de junio del 2019 (19).

## RESULTADOS

### Características de cobertura de TRR por país, accesibilidad y eficiencia

Debido a la epidemiología y organización sanitaria, se encontraron características específicas en relación con la oferta de servicios de TRR y su accesibilidad por cada país (cuadro 1).

**FIGURA 1.**
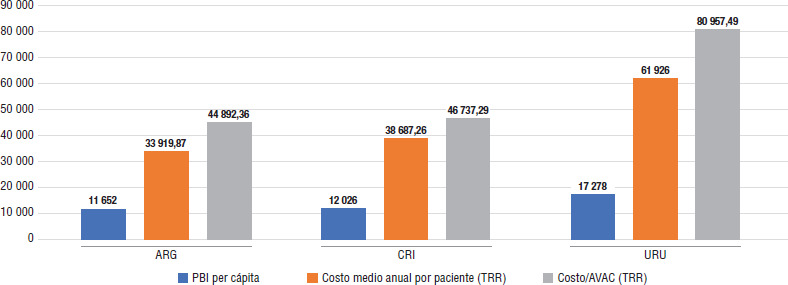
Comparación de los de datos económicos^a^ para la cobertura de terapias de reemplazo renal por país

a Expresados en dólares estadounidenses.

AVAC, años de vida ajustados por calidad; TRR, terapias de reemplazo renal; PBI, producto bruto interno; ARG, Argentina, CRI, Costa Rica; URU, Uruguay.

De manera complementaria, se relevaron los resultados económicos y sanitarios globales de cada país en función del PBI per cápita y el gasto destinado a las TRR, buscando mostrar lógicas sobre la priorización de TRR en función de la capacidad económica y las condiciones organizacionales (figura 1).

**CUADRO 1.** Características epidemiológicas, organizativas y económicas por país

**Características**	**Argentina**	**Costa Rica**	**Uruguay**
Habitantes (millones)	41,4	4,9	3,4
Prevalencia de TRR (pmp)	860	401	1 086
Total de pacientes con TRR	35 625	1 949	3 703
Prevalencia de TX (%)	23,0	59,4	29,7
Prevalencia de HD (%)	72,8	10,1	62,4
Prevalencia de DP (%)	4,2	30,5	6,1
Nefrólogos (pmp)	27	5	51
Financiamiento de las TRR	30% público + 70% seguro social	Único (solo sector público: CCSS)	Único (solo sector público: FNR)
Oferta TRR	Sector privado (90%)	Solo sector público	Sector privado (90%)
PBI per cápita (2019)	U$S 11 652	U$S 12 026	U$S 17 278
Gasto per cápita en TRR	U$S 29,17	U$S 15,51	U$S 67,31

TRR, terapia de reemplazo renal; TX, trasplante; DP, diálisis peritoneal; HD, hemodiálisis; CCSS, Caja Costarricense del Seguro Social; FNR, Fondo Nacional de Recursos; PBI, producto bruto interno; U$S, dólares estadounidenses; pmp, por millón de población.

### Costos relativos de las TRR por país

**FIGURA 2.**
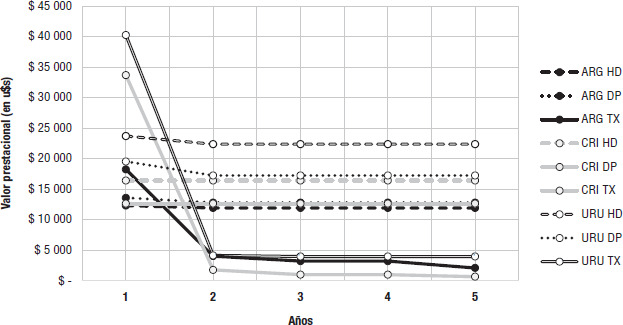
Costo anual^a^ de cada terapia de reemplazo renal por país

a A cinco años, expresados en dólares estadounidenses.

A partir de los costos directos de las prestaciones relacionadas a cada TRR, se evidenció una clara dispersión y asimetría entre los valores de los países. En la figura 2 se muestra el gasto por cada TRR y país, estimado a 5 años de seguimiento.

En todos los casos se observa el alto costo inicial del TX, que cae luego del segundo año. Las modalidades dialíticas presentan un punto levemente elevado el primer año (asociado a mayor morbimortalidad) y luego se mantienen fijas. En Argentina, ambas modalidades tienen casi el mismo valor, y la DP es, incluso, levemente más costosa.

### Punto de equilibrio entre el trasplante y las técnicas dialíticas

**FIGURA 3.**
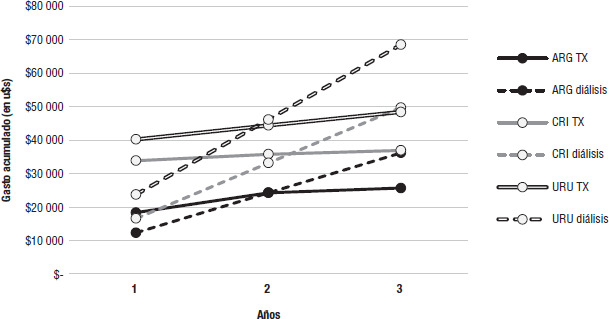
Comparación del punto de equilibro a tres años entre el trasplante y las técnicas dialíticas por país

ARG, Argentina; CRI, Costa Rica; URU, Uruguay; TX, trasplante.

En cada país existieron diferencias sobre los montos y los momentos en los que se alcanza el punto de equilibrio entre el gasto en TX frente a ambas modalidades dialíticas tomadas en conjunto (cuadro 2 y figura 3).

En todos los casos, el TX demuestra ahorrar costos. La neutralización de gastos ocurre de manera temprana, pero en diferentes momentos en cada país (por dispersión de precios relativos de compra de servicios); luego, los gastos acumulados en técnicas dialíticas crecen despegándose del TX en todos los modelos.

### Análisis de costo-efectividad

A partir del caso base ajustado a la prevalencia actual de cada método por país, se buscaron las alternativas que, al incrementar la prevalencia de las modalidades más eficientes (TX y DP), permitan conseguir ahorros de costos o incrementar los AVAC conseguidos dentro del umbral definido. En el cuadro 3 se ordenan los países y las alternativas de difusión de las diferentes TRR en términos de iniciativas CE. De manera secuencial, se dejaron en condición fija cada TRR, explorando la variación necesaria de las restantes para mejorar la eficiencia. La información se complementó en cada caso consignando la cantidad de pacientes nuevos por la TRR más eficiente, el número estimado de muertes evitables y los AVAC conseguidos con cada estrategia, así como los costos globales del sistema para cubrir estos tratamientos.

ARG, Argentina; CRI: Costa Rica; URU: Uruguay; HD, hemodiálisis; TX, trasplante; DP, diálisis peritoneal.

**CUADRO 2.** Punto de equilibrio por país

**Punto de equilibrio**	**Tiempo**	**Monto (U$S)**
Argentina	21 meses	21 439,79
Costa Rica	26 meses	35 998,70
Uruguay	23 meses	43 994,31

U$S, dólares estadounidenses.

**CUADRO 3.** Análisis de estrategias de costo-efectividad por país^a^

**País**	**Prevalencias**		**Pacientes inicio**	**Nuevos pacientes**	**Ahorro de costos** ^b^	**Incremento de costos** ^b^	**Muertes evitadas**	**Costo por cada muerte evitada** ^b^	**Total de AVAC**	**AVAC extra**	**Costo por cada AVAC extra** ^b^	**Gasto total en TRR** ^b^	**Costo por AVAC total en TRR**
	HD móvil	72,7%	25 899										
	**DP fijo**	**4,2%**	**1496**		$ 141 756	-----	20	-----	79 242	74	-----	$ 1 113 775 721	$ 14 055
	TX móvil	**23,1%**	8229	**36**									
	**HD fijo**	**72,8%**	**25935**										
**Argentina**	DP móvil	4,1%	1461		$ 673 570	-----	10	-----	79 208	41	-----	$ 1 113 243 908	$ 14 054
	TX móvil	**23,1%**	8229	**36**									
	HD móvil	56,0%	19 950										
	DP móvil	**21,0%**	7481	**5985**	-----	$ 89 116 581	1693	$ 52 638	84 797	5630	$ 15 828	$ 1 203 034 060	$ 14 187 Dominancia parcial^c^
	**TX fijo**	**23,0%**	**8194**										
	HD móvil	8,9%	98										
	**DP fijo**	**30,4%**	**333**		-----	$ 1 103 636	5	$ 220 727	6995	25	$ 44 145	$ 89 635 574	$ 12 814
	TX móvil	**60,7%**	665	**12**									
	**HD fijo**	**10,0%**	**110**										
**Costa Rica**	DP móvil	28,6%	313		-----	$ 991 827	6	$ 165 304	6993	23	$ 43 122	$ 89 523 765	$ 12 801
	TX móvil	**61,4%**	673	**20**									
	HD móvil	10,0%	110										
	DP móvil	30,4%	333	**20**	-----	$ 1 568 269,34	0	-----	0	0	-----	$ 90 100 207	Dominada^c^
	**TX fijo**	**59,6%**	**653**										
	HD móvil	63,0%	2333										
	**DP fijo**	**6,1%**	**226**		-----	$ 4 827 680	15	$ 321 845	9825	60	$ 80 461	$ 249 750 148	$ 25 419
	TX móvil	**30,9%**	1144	**44**									
	**HD fijo**	**64,2%**	**2377**										
**Uruguay**	DP móvil	4,2%	156		-----	$ 4 547 509	15	$ 303 167	9822	57	$ 79 780	$ 249 469 976	$ 25 399
	TX móvil	**31,6%**	1170	**70**									
	HD móvil	61,0%	2259										
	DP móvil	**9,3%**	344	**118**	-----	$ 5 308 915	14	$ 379 208	9830	66	$ 80 438	$ 250 231 383	$ 25 455 Dominancia parcial ^c^
	**TX fijo**	**29,7%**	**1100**										

TRR, terapias de reemplazo renal, HD, hemodiálisis; DP, diálisis peritoneal.

a Para cada país aparecen las tres opciones de ajuste: cuando una opción de TRR está en **negrita** (fija), la de color **rojo** muestra la opción más eficiente. Aumentar el trasplante en Argentina incluso genera ahorro de costos: el resto de los escenarios muestran alternativas que incrementa los costos, pero manteniéndose dentro del umbral de costo-efectividad definido.

b Expresado en dólares estadounidenses.

c La condición de dominancia se refiere a la identificación de la opción más desfavorecida en términos de costo efectividad al ser comparada con las otras opciones en evaluación.

Para el caso de Argentina, considerando la escasa diferencia relativa entre el costo de TX y las modalidades dialíticas, el incremento leve de la tasa de donación (0,1% = 36 nuevos trasplantes/año) se traduce en ahorro de costos. Cuando la proporción de TX se mantiene fija, las ganancias en muertes evitadas y AVAC dentro de la ICER límite se consiguen de manera directa al promover la DP. Una situación similar ocurre con Uruguay, aunque en este caso, por diferencia amplia de costos, la prevalencia de trasplante debe incrementarse en mayor proporción (1-2% = 44-70 nuevos trasplantes/año) y sin conseguir ahorro de costos; otra estrategia CE consistiría en incrementar la DP en más 50%, pasando 118 pacientes/año desde HD a DP.

Por último, para la situación de Costa Rica, la alta prevalencia actual de TX y las características de costo y calidad de vida del método limitan cualquier aporte marginal de beneficios a partir de las técnicas dialíticas: la ampliación de oferta de las mismas no genera beneficios en ahorro de costos ni ganancia de AVAC o muertes evitadas, y su inclusión obedecerá a necesidades poblacionales o epidemiológicas y no a criterios de eficiencia (con prevalencia de DP debido a que tiene menor costo que la HD) (cuadro 3).

## DISCUSIÓN

Las EES aplicadas a las TRR deben considerar aspectos particulares propios de la complejidad de estos tratamientos, que dependen en gran medida de las características organizacionales y económicas de los sistemas de salud donde se implementan (8). En el caso particular de este estudio sobre tres países latinoamericanos, pueden observarse asimetrías de accesibilidad y desarrollo de las TRR: en un análisis general, la compra de servicios con incentivo a la oferta del sector privado podría influir en una mayor disponibilidad de especialistas, más acceso a técnicas dialíticas y mayor gasto, con incremento en la prevalencia global de las TRR (11). Por otra parte, el sector público, mediante la integración del financiamiento y la prestación de servicios, tendría una limitante en la accesibilidad, pero lograría un uso más eficiente de los recursos invertidos (cuadro 1). Estas características organizativas parecen trasladarse a los resultados económicos y sanitarios en función del PBI per cápita y el gasto destinado a las TRR (19), evidenciando esta lógica particular de cada país en función de sus capacidades económicas y organizacionales.

En todos los casos, la promoción del TX se manifiesta como una estrategia que ahorra costos, aunque el momento donde se alcanzan los puntos de equilibrio depende de los precios relativos y accesibilidad a prestaciones por país. Estas diferencias ponen en evidencia la necesidad de determinar diferentes umbrales CE por país y el aporte que pueda ofrecer cada TRR en términos de eficiencia en el gasto (cuadro 2).

En el caso de Costa Rica, con alta prevalencia relativa de inicio en TX, el espacio de mejora de resultados en términos de CE por una mayor difusión de terapias dialíticas es marginal o inexistente; la incorporación de estas alternativas estará dada por la necesidad sanitaria (la DP es más eficiente, en términos de precios corrientes, que la HD). Por otra parte, Argentina y Uruguay presentan más margen de mejora en su perfil de CE si logran incrementar tanto sus tasas de donación con acceso al TX como la proporción de pacientes en DP.

En relación con los otros países, Costa Rica presentaría un margen presupuestario razonable de adjudicación de recursos para ampliar el acceso a las TRR, mientras que Argentina y Uruguay presentan una accesibilidad adecuada, aunque enfrentan el desafío de mejorar la oferta de DP e incrementar las tasas de donación para mantener su nivel de eficiencia actual.

Con base en los datos estimados de costos y variación clínica, la herramienta elaborada permite estimar la proporción de difusión de distintas estrategias dentro de un marco de costo-efectividad, como instrumento adicional para la toma de decisiones. Incluso en una temática compleja y de alto impacto como las TRR, el trabajo colaborativo liderado por una institución como la OPS (14) ha generado de manera consensuada un bien público en salud, accesible para sumar información de manera sistematizada y obtener comparaciones con base racional en la Región. La información fue relevada con bajo nivel de sesgo mediante la participación de equipos clínicos y económicos de los países participantes, que volcaron sus datos en el modelo de manera independiente, lo que aumenta la fortaleza de los resultados.

Cada país obtuvo beneficios adicionales y específicos durante la investigación: Argentina explicitó los umbrales y precios por cada financiador, Costa Rica desarrolló un microcosteo actualizado en TRR con evaluación de accesibilidad a los tratamientos, y Uruguay consiguió una integración de los costos en todas las etapas en TRR y definió los umbrales para una negociación futura con los prestadores.

Por último, los resultados sustentan las estrategias planteadas en el Plan Regional de Donación y Acceso Equitativo a Trasplantes 2019-2030 de la OPS, para promoción de la donación, la implementación de redes organizadas, el financiamiento de las etapas del TX incluida la inmunosupresión, y la disponibilidad de registros sobre donación y trasplante en pos de mejorar la equidad en el acceso.

## Conclusiones

Aún con limitaciones relativas a la diversidad de datos relevados (solo abordados a través de un análisis determinístico univariante), este estudio sobre la difusión de diferentes alternativas de TRR en tres países latinoamericanos confirma algunas características importantes. Se evidencia que la promoción del TX es una estrategia que ahorra costos, aunque los puntos de equilibrio variables muestran la necesidad de determinar diferentes umbrales de CE por país. En Argentina y Uruguay, la indicación y administración de TRR puede mejorar su eficiencia incrementando tanto la donación de órganos para TX como la proporción de pacientes en DP, mientras que en Costa Rica (con una prevalencia de trasplantes y un margen presupuestario adecuado), la incorporación de DP, HD o ambas estará dada por la demanda y la incidencia de nuevos pacientes con ERCT. La herramienta de análisis elaborada a partir del apoyo estratégico de la OPS adquiere características de bien público en salud, y permite incorporar información nueva accesible y comparada para mejorar la toma de decisiones.

## Declaración.

Las opiniones expresadas en este manuscrito son responsabilidad del autor y no reflejan necesariamente los criterios ni la política de la *RPSP/PAJPH* y/o de la OPS.
